# Caveolin-1 mediates soft scaffold-enhanced adipogenesis of human mesenchymal stem cells

**DOI:** 10.1186/s13287-021-02356-z

**Published:** 2021-06-14

**Authors:** Shiqi Xiang, Zhong Li, Madalyn R. Fritch, La Li, Sachin Velankar, Yuwei Liu, Jihee Sohn, Natasha Baker, Hang Lin, Rocky S. Tuan

**Affiliations:** 1 Center for Cellular and Molecular Engineering, Department of Orthopaedic Surgery, University of Pittsburgh School of Medicine, Pittsburgh, Pennsylvania USA; 2 Department of Orthopaedics, The Second Xiangya Hospital of Central South University, Changsha, Hunan, China; 3 Department of Chem/Petroleum Engineering and Mechanical Engineering & Materials Science, University of Pittsburgh Swanson School of Engineering, Pittsburgh, Pennsylvania USA; 4 McGowan Institute for Regenerative Medicine, University of Pittsburgh, Pittsburgh, Pennsylvania USA; 5 Present Address: Biogen, Boston, Massachusetts USA; 6 Present Address: Department of Oral Biology, University of Pittsburgh School of Dental Medicine, Pittsburgh, Pennsylvania USA; 7 Department of Bioengineering, University of Pittsburgh Swanson School of Engineering, Pittsburgh, Pennsylvania USA; 8 Present Address: Institute for Tissue Engineering and Regenerative Medicine, The Chinese University of Hong Kong, Shatin, Hong Kong SAR, China

**Keywords:** Mesenchymal stem cells, Substrate stiffness, Photocrosslinked gelatin hydrogel, Adipogenesis, YAP, CAV1

## Abstract

**Background:**

Human bone marrow-derived mesenchymal stem cells (hBMSCs) can differentiate into adipocytes upon stimulation and are considered an appropriate cell source for adipose tissue engineering. In addition to biochemical cues, the stiffness of a substrate that cells attach to has also been shown to affect hBMSC differentiation potential. Of note, most current studies are conducted on monolayer cultures which do not directly inform adipose tissue engineering, where 3-dimensional (3D) scaffolds are often used to create proper tissue architecture. In this study, we aim to examine the adipogenic differentiation of hBMSCs within soft or stiff scaffolds and investigate the molecular mechanism mediating the response of hBMSCs to substrate stiffness in 3D culture, specifically the involvement of the integral membrane protein, caveolin-1 (CAV1), known to regulate signaling in MSCs via compartmentalizing and concentrating signaling molecules.

**Methods:**

By adjusting the photo-illumination time, photocrosslinkable gelatin scaffolds with the same polymer concentration but different stiffnesses were created. hBMSCs were seeded within soft and stiff scaffolds, and their response to adipogenic induction under different substrate mechanical conditions was characterized. The functional involvement of CAV1 was assessed by suppressing its expression level using CAV1-specific siRNA.

**Results:**

The soft and stiff scaffolds used in this study had a compressive modulus of ~0.5 kPa and ~23.5 kPa, respectively. hBMSCs showed high viability in both scaffold types, but only spread out in the soft scaffolds. hBMSCs cultured in soft scaffolds displayed significantly higher adipogenesis, as revealed by histology, qRT-PCR, and immunostaining. Interestingly, a lower CAV1 level was observed in hBMSCs in the soft scaffolds, concomitantly accompanied by increased levels of Yes-associated protein (YAP) and decreased YAP phosphorylation, when compared to cells seeded in the stiff scaffolds. Interestingly, reducing CAV1 expression with siRNA was shown to further enhance hBMSC adipogenesis, which may function through activation of the YAP signaling pathway.

**Conclusions:**

Soft biomaterials support superior adipogenesis of encapsulated hBMSCs in 3D culture, which is partially mediated by the CAV1-YAP axis. Suppressing CAV1 expression levels represents a robust method in the promotion of hBMSC adipogenesis.

**Supplementary Information:**

The online version contains supplementary material available at 10.1186/s13287-021-02356-z.

## Background

Fat is the most abundant tissue of the human body. It provides structural protection, shapes a normal human appearance, and plays key roles in endocrine and metabolic functions [1]. Due to aging, trauma, or pathological conditions, one can suffer damage to or significant losses in adipose tissue, often requiring surgical interventions. Free-fat transplantation represents a classic approach for replacing lost or damaged adipose tissue, but is often limited by delayed neovascularization, graft volume shrinkage, and poor long-term survival [2]. In recent years, the emerging technologies of tissue engineering strategy, which typically involve the generation of new tissues through guided differentiation of cells encapsulated a 3-dimenstional (3D) scaffold, have presented alternative approaches for regenerating adipose tissue. Specifically, adult mesenchymal stem cells derived from the human bone marrow (hBMSCs) or fat, which have shown robust adipogenic potential upon stimulation, are the two most used cell sources in current adipose tissue engineering [3–5]. To generate tissues with sufficient volume for large defect repair, these cells are often combined with scaffolds that provide structural support as well as influence cell behavior through cell-matrix interactions. For example, robust adipogenesis was reported for hBMSCs seeded with gelatin-based scaffolds [6] and polylactic acid nanofibrous scaffold [7] upon treatment with adipogenic medium.

In addition to the inducing agents present in adipogenic medium, hBMSC adipogenesis is also affected by biomechanical signals [8–14]. Majumder et al. [15] reported that a soft substrate is better able to maintain the adipogenic differentiation ability of hBMSCs. Similar results were reported in another study, which demonstrated that a greater degree of adipogenesis occurs on softer matrices, as evidenced by the accumulation of lipid droplets [16]. Of note, most current studies investigating the response of hBMSCs to substrate stiffness were conducted on two-dimensional (2D) cultures [17]. Although this conventional culture condition allows easy cell manipulation and analysis, it does not assess 3D cultures commonly used in tissue engineering. Therefore, an increasing number of studies have recently been focusing on cell behavior in 3D culture environments. For example, Chaudhuri et al. seeded hBMSCs in alginate scaffolds and found that cells cultured in a softer alginate scaffold produced more oil drops and expressed higher levels of adipogenic genes than cells in stiff scaffolds [18]. Based on current studies, it is generally accepted that softer substrates enhance the level of adipogenesis, irrespective of 2D or 3D culture [19].

Integrins and associated molecules, specifically the YAP (Yes-associated protein) and its paralog TAZ (transcriptional co-activator with PDZ-binding motif), components of the Hippo signaling cascade, have been shown to play a role in the response of hBMSCs to substrate stiffness [20]. However, the outcome of differentiation also depends highly on the dimensionality of the culture system [21]. Interestingly, integrin endocytosis, one means of integrin regulation, is mediated by caveolin-1 (CAV1) coated-caveolae membrane rafts [22]. It has also been reported that CAV1 positively modulates YAP activity under 2D conditions [23]. To date, the interplay between CAV1 and YAP/TAZ during the cell’s response to substrate stiffness and adipogenic medium in the context of 3D culture conditions has not been fully studied.

Herein, we aim to examine the efficiency of hBMSC adipogenesis within soft and stiff hydrogels and investigate how CAV1 and YAP/TAZ regulate the response of hBMSCs to substrate stiffness. We aimed to test the hypothesis that a soft 3D scaffold would support a higher level of adipogenesis than a stiff one. To test this hypothesis, we first prepared photo-crosslinked hydrogels with the same gelatin concentrations but different stiffnesses by adjusting the time the scaffolds were cured under photo-crosslinking illumination. Then, the viability and morphology of hBMSCs within soft and stiff scaffolds were examined. After hBMSC-laden soft or stiff scaffolds were cultured in adipogenic medium for 2 weeks, the level of adipogenesis was examined by qRT-PCR, histology, and immunostaining. Lastly, we assessed the alterations of CAV1 and YAP levels in cells encapsulated in soft and stiff scaffolds during adipogenesis and then used siRNA to downregulate the expression levels of CAV1 to examine the role of CAV1 and YAP in regulating adipogenesis in the context of 3D culture.

## Methods

### Preparation of hydrogel scaffolds with different levels of stiffness

Gelatin scaffolds were fabricated using previously reported methods [24, 25]. Briefly, to synthesize the methacrylated gelatin (GelMA), bovine skin-derived gelatin (Sigma-Aldrich, St. Louis, MO) was dissolved in deionized H_2_O and then reacted with methacrylic anhydride (Sigma-Aldrich) overnight at 37°C. The reaction product was dialyzed against distilled water using a dialysis cassette (3.5K molecular weight cut-off membrane, ThermoFisher, Waltham, MA) for 5 days to remove the salts and methacrylic acid. The solution was then lyophilized, and the dried sponge was stored in a desiccator. To make the monomer solution, GelMA was dissolved into Hanks’ Balanced Salt Solution (HBSS, with Ca^2+^ and Mg^2+^; ThermoFisher) at 15% (w/v), and the photoinitiator, lithium phenyl-2,4,6-trimethyl-benzoyl phosphinate (LAP, Sigma-Aldrich, St. Louis, MO), was added at 0.15% (w/v).

To generate the hydrogel scaffolds, the GelMA/LAP solution was first poured into silicon molds, which had a cylindrical void space (2 mm height × 5 mm diameter). Then, polymerization was induced using a flashlight with a wavelength at 395 nm for 8 s or 2 min to generate soft or stiff scaffolds.

### Degradation behavior of hydrogels

The biodegradability of hydrogel scaffolds was evaluated by incubating gels in collagenase type 1 solution in phosphate-buffered saline (PBS) (0.05%, w/v) (Worthington-Biochemical Corporation, Lakewood, NJ) at 37°C with shaking at 50 rpm/min. At different time points, samples were taken, washed, and weighed. Biodegradability was quantified by determining the % remaining weight as follows:

$$ \%\mathrm{Weight}\ \mathrm{remaining}={\mathrm{W}}_{\mathrm{t}}/{\mathrm{W}}_0\times 100\%. $$

where W_0_ was the initial wet weight of the hydrogel scaffold and W_t_ is the weight at different time points (t).

### Mechanical test

The compressive moduli of hydrogel scaffolds created with different photo-illumination times were measured using a mechanical tester (Bose ElectroForce 3230 Series II, TA Instruments, New Castle, DE). Briefly, the hydrogels were subjected to 10% compression (0.2 mm) at 0.01 mm/s, and the linear portion of the stress-strain curve was used to calculate the compressive modulus of the scaffolds.

### Rheological analysis

The continuous and oscillatory shear measurements at small strain were used to characterize the viscoelastic properties of the hydrogel scaffolds. Both experiments were conducted using an Anton Paar MCR 302 rheometer (Ashland, VA). For test preparation, the scaffolds were placed between two profiled parallel plates with a diameter of 25 mm to prevent wall slip and were preheated at 37 °C to simulate body temperature. A pre-shear test was first run at a shear rate of 1   s^-1^ for 10 min for each sample. The shear rates for the continuous shear tests increased from 0.1 to 100 s^-1^, with a 1 min hold at each shear rate. In the oscillatory experiments, amplitude sweep tests were carried out with the strain ranging from 0.01% up to 100% at a constant frequency of 1 s^-1^.

### Harvest and expansion of human bone marrow-derived mesenchymal stem cells (hBMSCs)

According to an Institutional Review Board (IRB) exempted approval protocol (University of Washington), the surgical waste from total joint replacements was used for hBMSC isolation. The methods have been reported in our previous studies [24, 26]. Briefly, the femoral heads were flushed with a rinsing medium (α-MEM and 1% antibiotic-antimycotic, Invitrogen, Carlsbad, CA). The cell suspension was centrifugated, and the pellet was resuspended in the growth medium [GM, Dulbecco’s modified Eagle’s medium (DMEM; Gibco, Grand Island, NY) supplemented with 10 % (v/v) fetal bovine serum (FBS, Gemini Bio-Products, West Sacramento, CA) and 1× antibiotic-antimycotic (anti-anti; Gibco)] supplemented with 1.5 ng/mL fibroblast growth factor-2 (FGF-2; RayBiotech, Norcross, GA) and cultured in T150 flasks (Corning Inc., corning, NY)). Upon reaching 70–80% confluence, cells were detached by trypsin-0.25% ethylenediaminetetraacetic acid (ThermoFisher, Waltham, MA) and passaged. At passage 3 (P3), hBMSCs isolated from 5 male and 5 female donors were pooled. P5 cells were used in all experiments.

### In vitro culture of cell-laden gelatin scaffolds

To generate the two cell-laden hydrogel scaffolds with different stiffnesses, hBMSC pellets were resuspended in GelMA/LAP solution at a final density of 10 × 10^6^ cells/ml and then subjected to 8 s (soft) or 2 min (stiff) illumination using the method described above. The hBMSC-laden soft and stiff hydrogels were cultured in GM overnight and then maintained in adipogenic medium [27] (AM: α-MEM (Gibco) supplemented with 10% FBS, 1% antibiotics-antimycotics, 0.45 mM 3-isobutyl-1-methylxanthine (Sigma-Aldrich, St. Louis, MO), 0.1 μM dexamethasone (Sigma-Aldrich), 0.2 mM indomethacin (Sigma-Aldrich), and 1× insulin-transferrin-selenium (ITS, Invitrogen)).

### Analysis of cell morphology and cell viability in 3D hydrogels

Both cell morphology and cell viability were assessed after 1, 4, and 7 days of culture in AM. For morphological observation, the cell-laden scaffolds were fixed in 4% paraformaldehyde (PFA) aqueous solution (Fisher Scientific, Hampton, NH) for 2 h and permeabilized with 0.1% Triton X-100 (Sigma-Aldrich). Alexa Fluor 488 phalloidin (Invitrogen, Carlsbad, CA) was used to label cytoskeletal actin filaments, and Hoechst 33342 solution (Invitrogen, Carlsbad, CA) was utilized as nuclear counterstain. To assess cell viability, the LIVE/DEAD cell viability assay (Life Technologies, Carlsbad, CA) was used. Briefly, constructs were transferred into maintenance medium (phenol red-free DMEM (Gibco) containing 10% (v/v) FBS), containing calcein AM and ethidium homodimer-1, and then incubated at 37°C for 30 min. Images were acquired using an Olympus Fluoview 1000 confocal microscope (Center Valley, PA). *Z* stacks were acquired at optimal intervals (2 μm or 4 μm steps, 100–150 μm stack) as suggested by the software. NIH ImageJ software was utilized to analyze all the confocal stacks.

### Gene expression analysis

To isolate total cellular RNA, cell-laden scaffolds were transferred into a 1.5-ml RNase-free microtube and cells were lysed in QIAzol reagent, followed by RNA extraction using a RNeasy Plus Universal Kit (Qiagen, Germantown, MD). Reverse transcription was then carried out using the SuperScript® VILO^TM^ cDNA Synthesis Kit (Invitrogen) to obtain complementary DNA. qRT-PCR was then performed using SYBR Green chemistry and the QuantStudio 3 qRT-PCR system (Applied Biosystems, Foster City, CA). Relative gene expression was calculated using the comparative Ct (2^-ΔΔCT^) method, and the housekeeping gene glyceraldehyde-3-phosphatase dehydrogenase (*GAPDH*) was used as the endogenous control. The sequences of primers are listed in Table S1.

### Histology and immunostaining

The cell-laden 3D hydrogel constructs were fixed in 4% PFA and embedded in Cryo-gel (Leica Microsystems Inc, Chicago, IL). Blocks were sectioned at 6-μm thickness, and Oil Red O staining was performed to detect the lipid droplet [27]. The stained sections or cultures in 6-well plates were imaged with an EVOS M5000 Imaging System (ThermoFisher, Waltham, MA). To quantify the lipid content, cells on the culture plates were first washed exhaustively in distilled water and dried by placing it at 32 °C for 40 min [28]. Isopropyl alcohol was added to elute the dye from the cells and dye intensity was estimated spectrophotometrically based on A_510_ (Microplate Reader, BioTek, Winooski, VT).

BODIPY 493/503 (10 μg/ml, Invitrogen, D3922) was also used for lipid detection. Cryosections were washed with 1% (v/v) PBS-T solution for 10 min and then incubated with BODIPY solution for 30 min. Hoechst 33342 (ThermoFisher, H3570) was used as nuclear counterstain.

For immunofluorescence (IF), the 4% PFA-fixed cryosections were first blocked in 10% goat serum (ab7481, Abcam, Cambridge, UK) in PBS for 1h and incubated with primary antibodies against C/EBP-α (8178s, Cell Signaling Technology, Danvers, MA, 1:100 dilution) or CAV1 (3238s, Cell Signaling Technology, 1:400 dilution) overnight at 4°C. The information on the primary antibodies used in this study is listed in Table S2. For secondary antibodies, a goat anti-rabbit IgG (Alexa Fluor 488; Abcam, 1:500 dilution) was utilized. Alexa Fluor 568 conjugated phalloidin (Invitrogen, A12380, 1:200 dilution) was used to stain actin fibers. The samples were then mounted with a 4′,6-diamidino-2-phenylindole (DAPI)-containing antifade medium (Vector Laboratories, Burlingame, CA). An Olympus IX81 inverted microscope (Olympus, Waltham, MA) and EVOS M5000 Imaging System (ThermoFisher) were used to image the stained sections.

### Western blot analysis

Constructs were washed in PBS for 3 times and then pulverized in RIPA buffer (Sigma Aldrich) supplemented with 1% (v/v) protease and phosphatase inhibitor cocktail (ThermoFisher). After centrifugation, protein concentrations of the supernatants were determined using the BCA protein assay kit (Thermo Scientific BCA Protein Assay Kit). After thermal denaturation and reduction in Laemmli buffer containing β-mercaptoethanol (10% (v/v) (BioRad; Hercules, California), the proteins were electrophoretically fractionated in a 4–12% Bis-Tris polyacrylamide gel (Invitrogen™, NP0326BOX) and transferred onto PVDF membranes (0.2 μm). After being blocked in 3% non-fat milk (BioRad) in TBST (0.1% Tween-20 in TBS) for 1.5 h at room temperature, the membranes were incubated at 4°C overnight with primary antibodies against target proteins (diluted in 1% non-fat milk in TBST; see information in Table S2). After washing in TBST for 5 times, the membranes were incubated with horseradich peroxidase (HRP)-conjugated secondary antibodies (GENA934-1ML, Sigma-Aldrich, 1:2000 dilution) for 1.5 h at room temperature and then with SuperSignal West Femto Maximum Sensitivity Substrate solution (ThermoFisher). The blots were imaged using the ChemiDocTM Touch Imaging System (Bio-Rad). Quantification of the blot images was conducted using NIH ImageJ.

### siRNA treatment

siRNA transfection was performed as described previously [29]. Briefly, small interfering RNA (CAV1, Invitrogen) and negative control siRNA (Silencer Negative Control No. 1 siRNA, Invitrogen) were incubated with Lipofectamine™ RNAiMax reagent (Invitrogen) respectively in Opti-MEM medium for 5–10 min. The mixture was added to cell culture and incubated at 37°C for 48 h. Transfection efficiency was assessed using Western blot and qRT-PCR. The transfected cells were subjected to 3D culture using the same conditions described above.

### Statistical analyses

Statistical analysis was carried out using GraphPad Prism 9 (GraphPad, San Diego, CA). All data are presented as means and 95% confidence intervals for analyzing the correlation of gene expression. Mean differences between the two groups were assessed with Student’s *t* test. Analysis of variance (ANOVA) was used to analyze results among multiple groups. *p* values less than 0.05 were considered statistically significant, and depicted in figures as *p < 0.05, **p < 0.01, ***p < 0.001, and ****p < 0.0001.

## Results

### Soft and stiff scaffolds are fabricated from GelMA with a same concentration

**Fig. 1:**
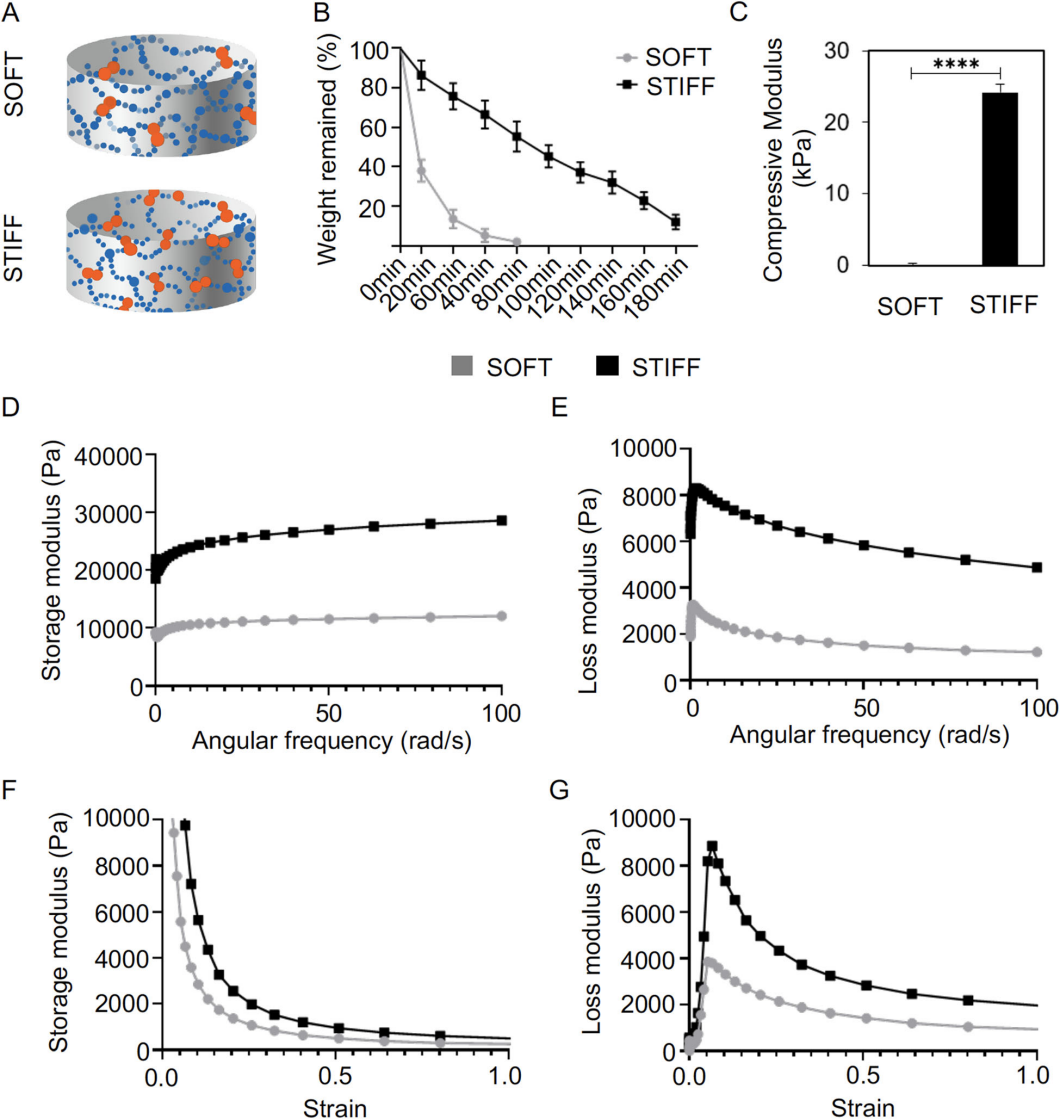
Characterization of soft and stiff scaffolds used for hBMSC culture. **a** Schematic illustration of the soft and stiff gelatin scaffolds, which had the same gelatin concentrations but different crosslinking degrees. **b** Degradation test of scaffolds in collagenase solution. Scaffolds were harvested at different time points and weighed. *N*= 10. **c** Compressive modulus of soft and stiff scaffolds. *N* = 3. ****, *p*<0.0001. **d**, **f** Storage modulus and **e**, **g** loss modulus of soft and stiff scaffolds in the continuous shear tests (**d**, **e**) or the oscillatory shear tests (**f**, **g**)

In this study, GelMA was used to fabricate scaffolds with different stiffnesses. The degree of crosslinking was controlled by adjusting the light exposure time (8 or 120 s), to create soft and stiff scaffolds, respectively (Fig. 1a). In both scaffolds, the gelatin concentration was the same (15% w/v). Upon collagenase treatment, soft scaffolds displayed faster degradation rates. Thus, in less than 80 min, the soft scaffolds were completely degraded (Fig. 1b). In contrast, it took more than 180 min to degrade stiff scaffolds. As expected, soft scaffolds had a significantly lower average compressive modulus of 0.5 kPa, while stiff scaffolds had an average compressive modulus of 23.5 kPa (Fig. 1c).

Figures 1d, e shows that in the continuous shear tests, as the crosslinking time increased from 8 s to 120 s, the storage and loss moduli of the hydrogel both significantly increased in the frequency range of 0.1–100 s^-1^. The storage and loss moduli values relate to the hydrogel’s elastic and viscous behaviors, respectively. For both sample groups, the storage moduli values were substantially larger than the loss storage values, indicating that soft and stiff hydrogels both displayed mainly elastic behavior. In the oscillatory shear tests (Fig. 1f, g), larger storage and loss moduli values were also observed for the stiff hydrogels when strains of 0.01 to 100% were applied, suggesting a larger resistance to deformation caused by forces exerted by the encapsulated cells. Therefore, cells gown in the stiff hydrogels were expected to experience a larger constraint by the local matrix than those in the soft hydrogels. In addition, both soft and stiff hydrogels showed a drastic decrease in storage moduli values in the strain range of 0.01–20%, indicating their decreased ability to recover from larger deformations.

### hBMSCs display different morphologies when cultured in soft and stiff scaffolds

**Fig. 2:**
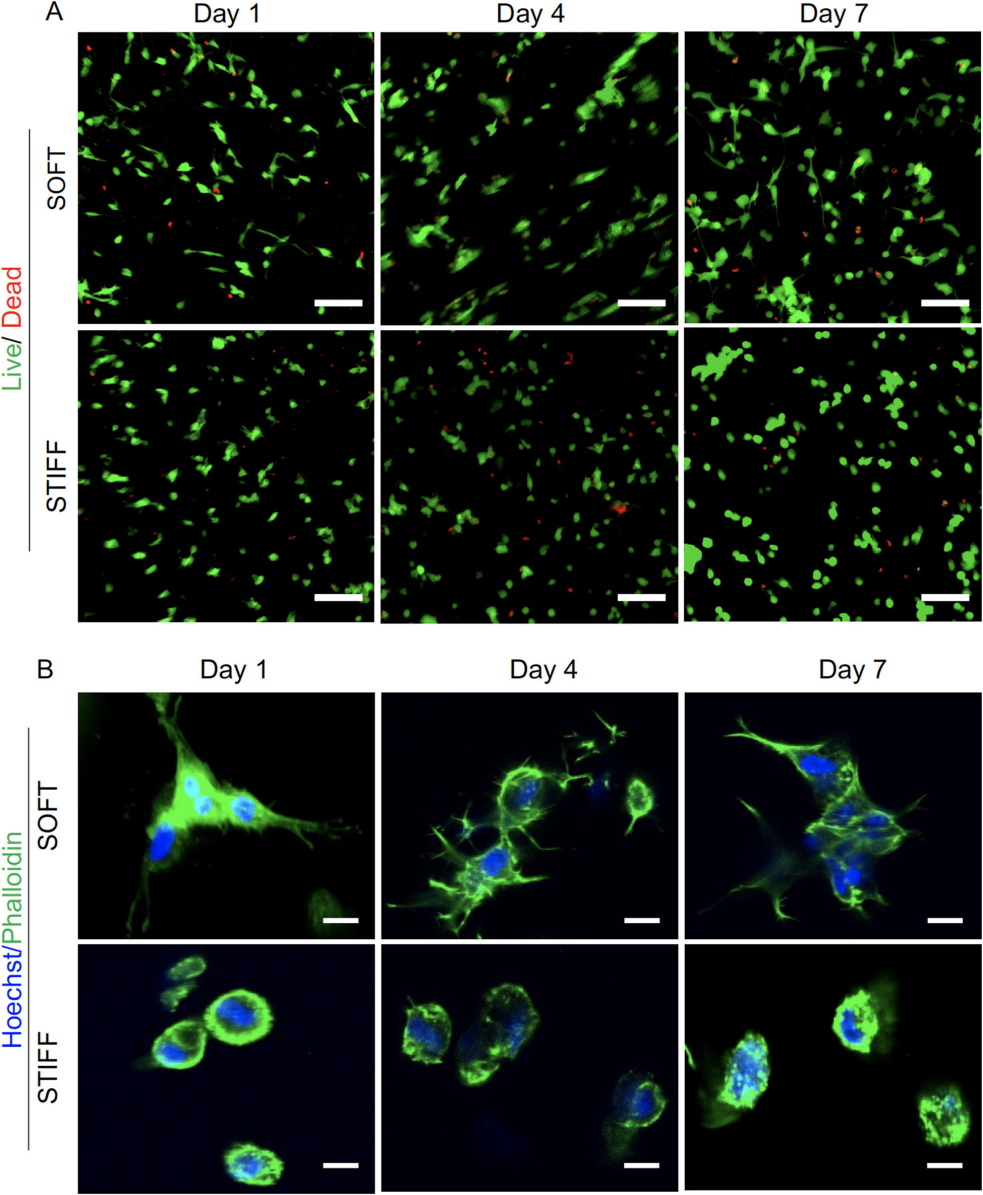
Viability and morphology of hBMSCs cultured in soft and stiff scaffolds. **a** Live/Dead staining of hBMSCs cultured for 1, 4, and 7 days. Green = live cells; Red = dead cells; Bar = 100 μm. **b** Representative higher magnification imaging by confocal microscopy showing cell morphology. Bar = 20 μm


hBMSCs encapsulated in both soft and stiff scaffolds were assessed for cell viability and morphology on days 1, 4, and 7. As shown in Fig. 2a, hBMSCs displayed high viability (>90%) in both scaffolds at all time points tested. Interestingly, phallodin cytoskeletal staining showed that cells within soft scaffolds spread and extended in all directions (Fig. 2b, Video S1), whereas cells within stiff scaffolds displayed a rounder morphology with limited development of actin filaments (Fig. 2b, Video S2).

**Additional file 2.**



**Additional file 3.**


### Culturing within soft scaffolds results in higher level of hBMSC adipogenesis

**Fig. 3:**
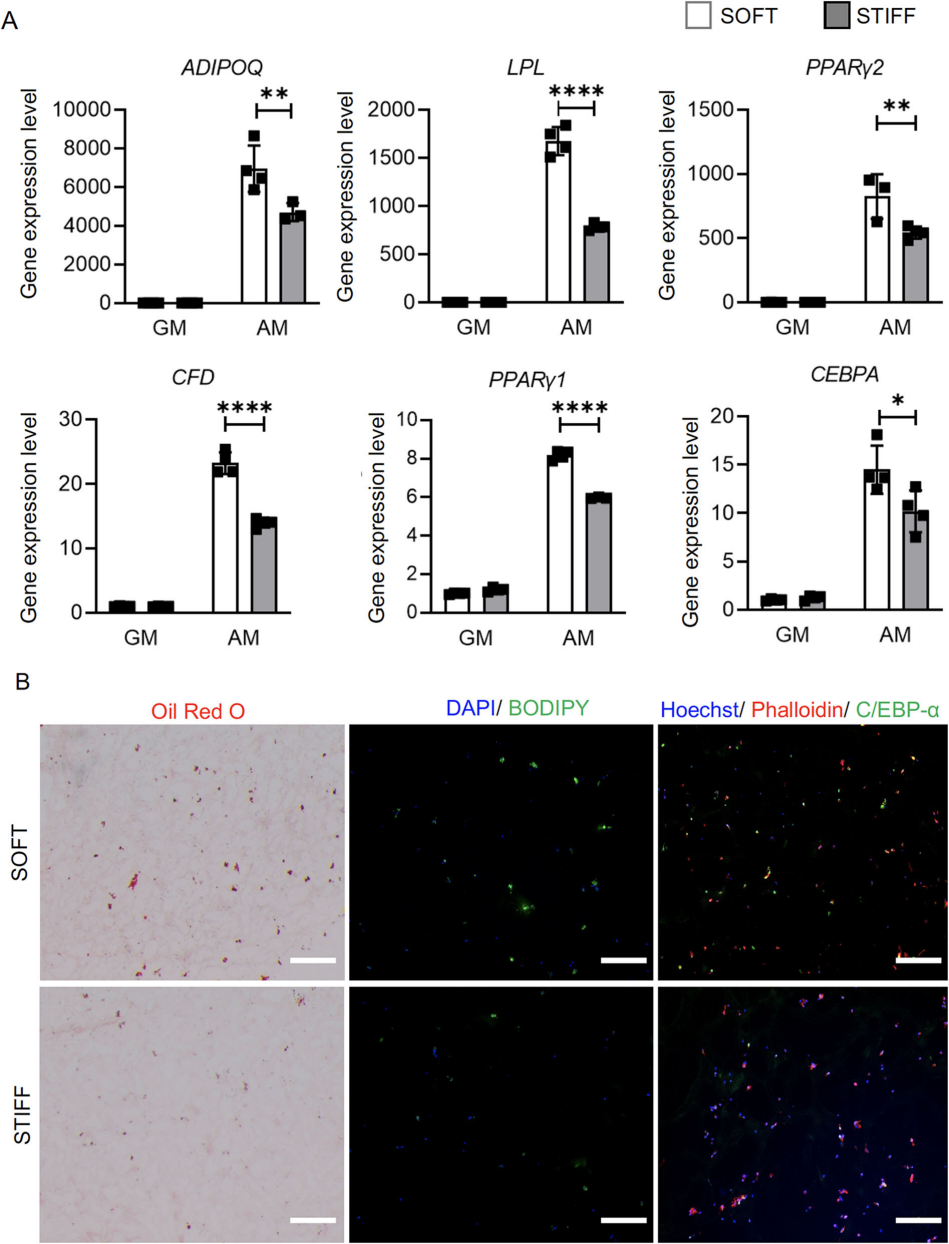
Assessment of adipogenesis in hBMSC-laden scaffolds. **a** Relative expression levels of adipogenic marker genes in hBMSCs cultured under adipogenic conditions (AM) in soft or stiff scaffolds. Data are normalized to that from the Soft-GM group (cultured under control growth conditions). *N* = 4. *p<0.05, **p<0.01, ****p<0.0001. GM: growth medium, AM: adipogenic medium. **b** Oil Red O staining, BODIPY staining, and C/EBP-α immunofluorescence to assess adipogenesis. Bar = 100 μm

hBMSC-laden soft and hard scaffolds were cultured in adipogenic medium for 14 days. As shown in Fig. 3a, all tested adipogenic genes were upregulated upon adipogenic stimulation. When compared to those in stiff scaffolds, cells within soft scaffolds showed higher expression levels of adipogenic genes, as well as produced more histologically detectable lipid droplets, C/EBP-α protein and BODIPY staining (Fig. 3b). A similar trend was observed on day 7 as well (Figure S1), which collectively implied a higher level of adipogenesis in soft scaffolds.

### hBMSCs cultured in soft scaffolds express lower level of CAV1

**Fig. 4:**
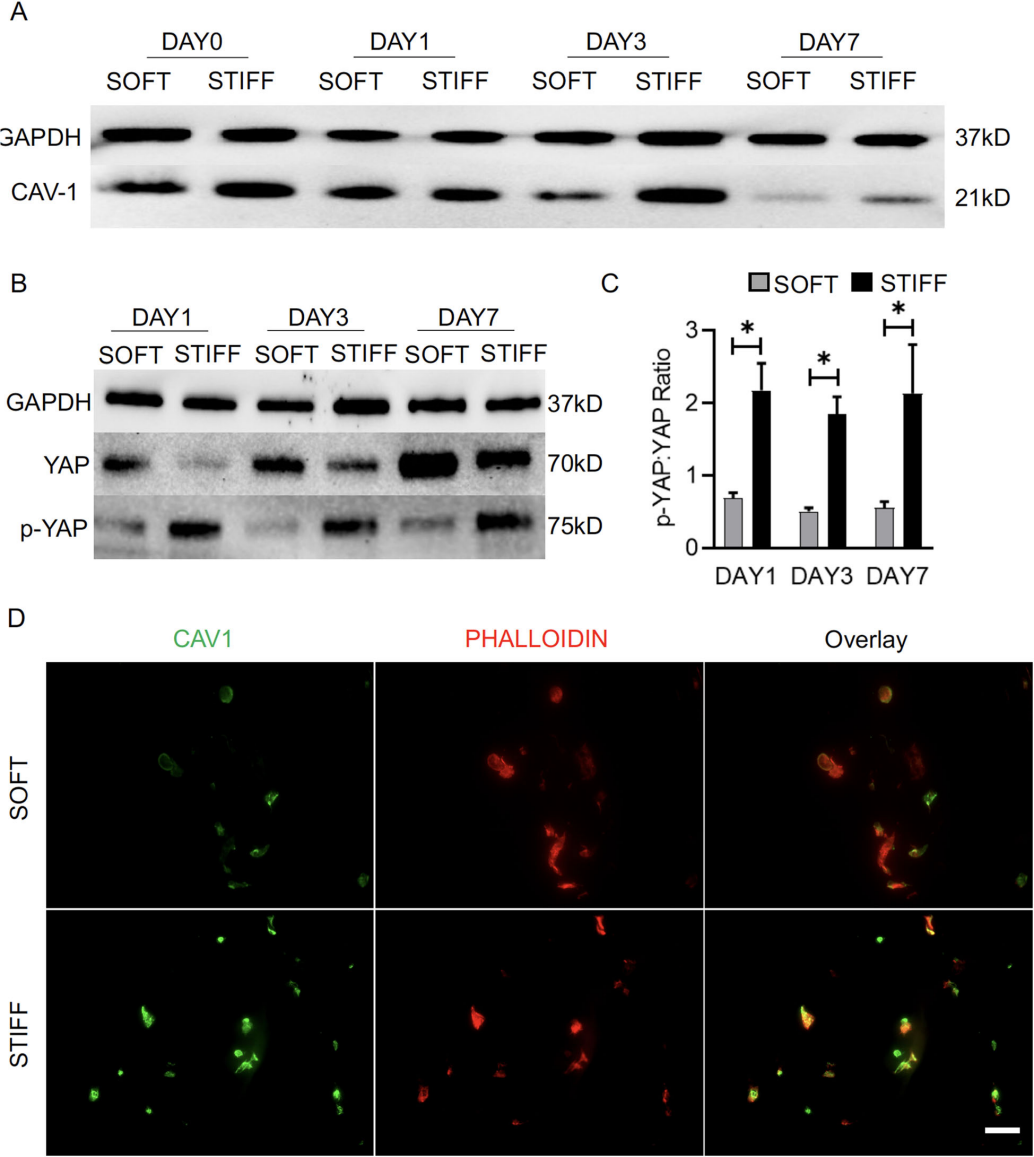
Examination of CAV1 and YAP levels in hBMSCs during adipogenesis in soft and stiff scaffolds. **a**, **b** Protein levels of CAV1, YAP, and p-YAP in hBMSCs were examined after 1, 3, and 7 days of adipogenic culture in soft or stiff scaffolds. **c** Semi-quantitative ratios of p-YAP:YAP on different culture days based on immunoblot band intensities. **d** CAV1 immunofluorescence on day 7. Bar = 50 μm. *p<0.05. *N* = 3

We then used immunofluorescence and Western blot to examine the expression level of CAV1. As shown in Fig. 4a, d, lower CAV1 levels were observed in softer scaffolds than in stiff scaffolds at all tested time points, except on day 1. In addition, as shown in Fig. 4b, c, higher levels of YAP and lower levels of YAP phosphorylation (p-YAP:YAP) were observed in cultures maintained in the soft scaffolds, suggesting that YAP activation is higher in soft cultures. In summary, CAV1 and YAP are closely related in regulating cellular responses to stiffness and adipogenic stimulation.

### Knockdown of *CAV-*1 results in enhanced adipogenesis

**Fig. 5:**
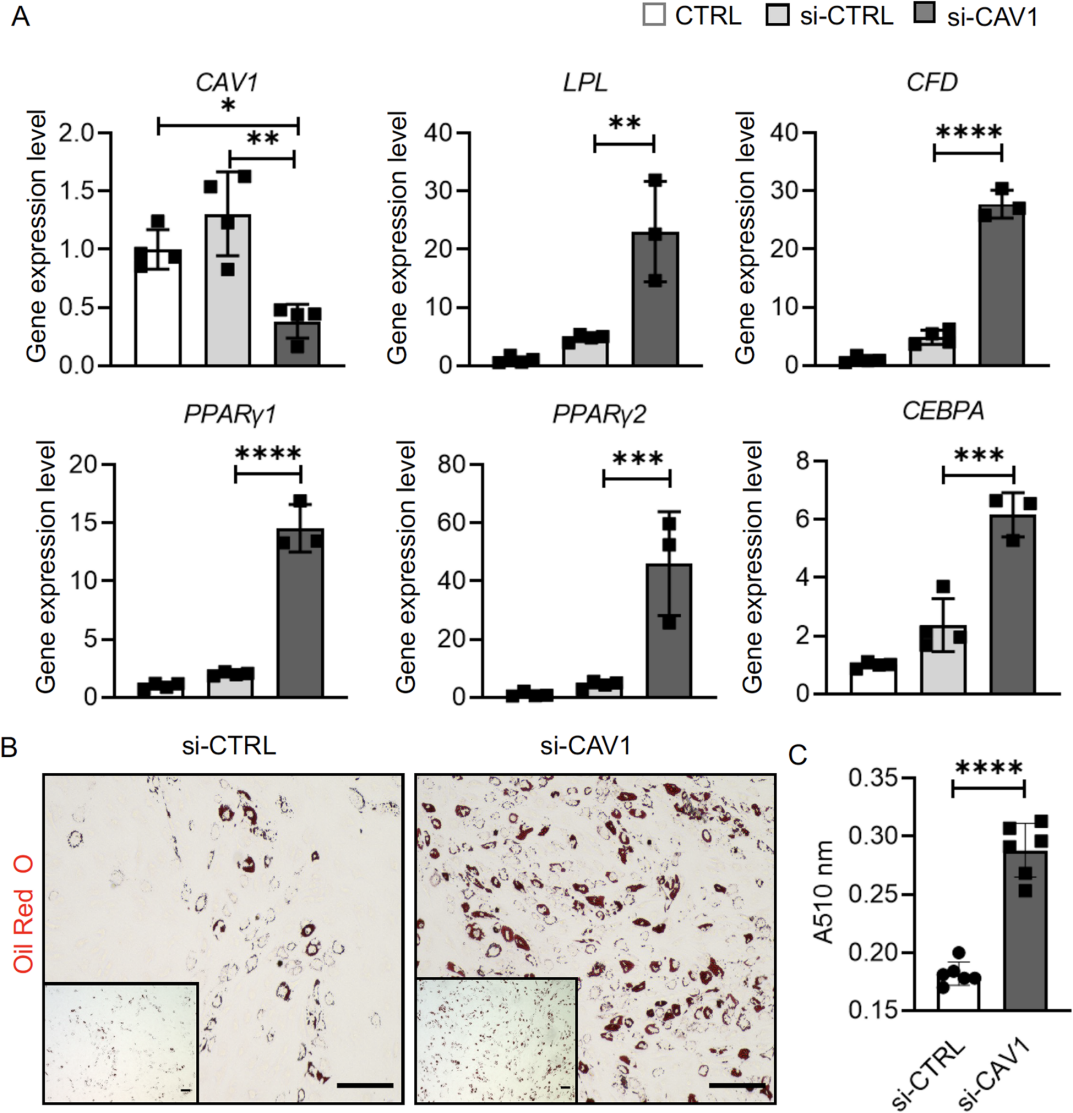
Influence of *CAV1* gene expression knockdown on adipogenesis in monolayer culture maintained in adipogenic medium. Control siRNA (si-CTRL) and CAV1 siRNA (si-CAV1) were used. **a** Relative expression levels of *CAV1* and adipogenic marker genes. Data are normalized to that in the CTRL group, which was not treated with siRNA or transfection agents. *N* = 4. **b** Oil Red O staining. Bar = 100 μm. **c** Lipid staining was extracted and quantified spectrophotometrically. *p<0.05, **p<0.01, ***p<0.001, ****p<0.0001. *N* = 6

To understand the function of CAV1, siRNA was used to reduce the expression levels of *CAV1*. Transfection with *CAV1* siRNA resulted in an ~8-fold reduction in the expression levels of *CAV1* 48 h after transfection (Figure S2). This knockdown effect lasted up to 2 weeks (Fig. 5a). When compared to the untreated control (CTRL group) or control siRNA-treated cells (si-CTRL group), hBMSCs treated with *CAV-1* siRNA (si-CAV1 group) displayed higher levels of adipogenesis upon adipogenic stimulation in 2D culture, revealed by qRT-PCR and Oil Red O staining (Fig. 5a, b). In particular, cells in the si-CAV1 group generated ~50% more oil droplet staining than those in the si-CTRL group (Fig. 5c).

**Fig. 6:**
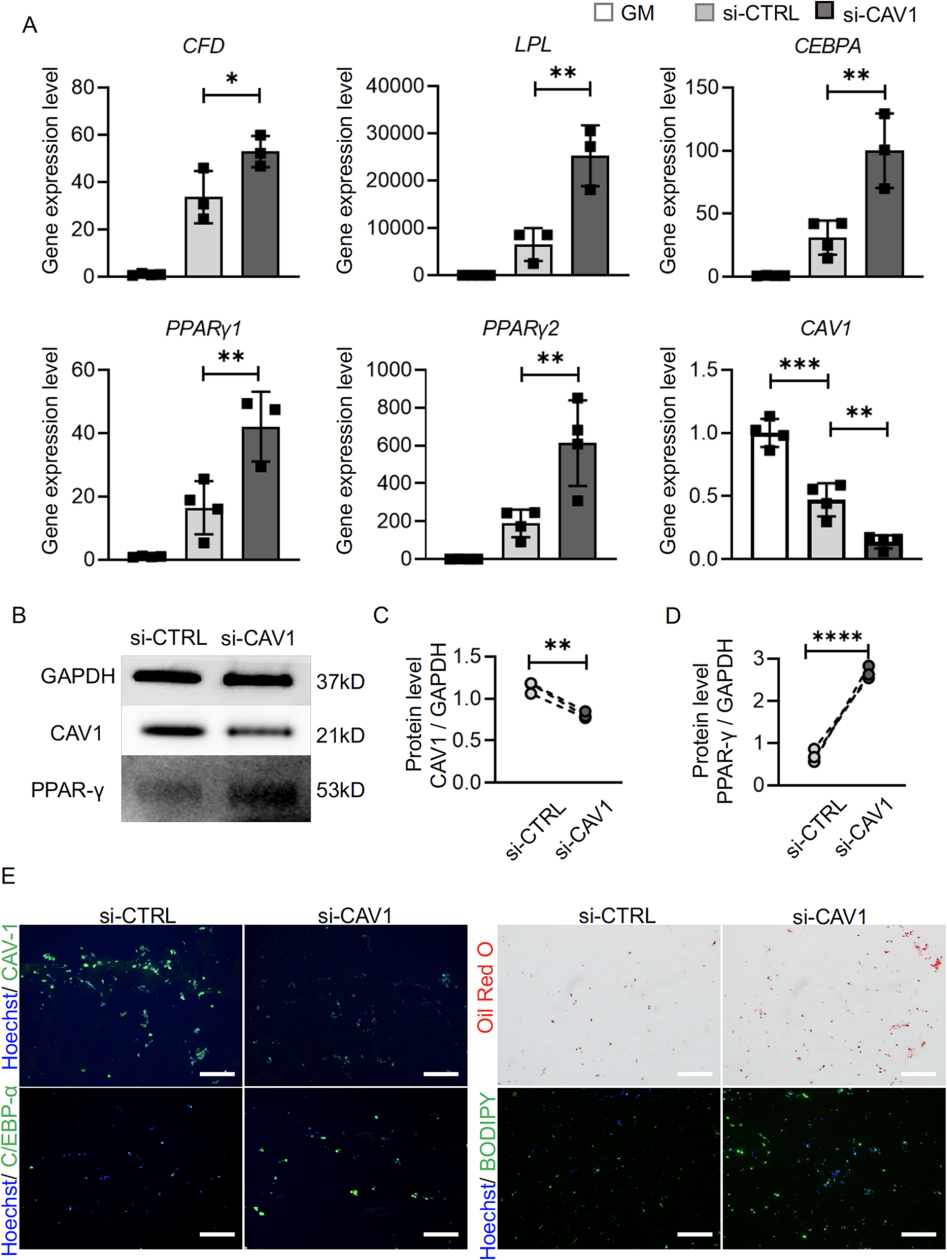
Influence of *CAV1* gene expression knockdown on hBMSC adipogenesis in soft and stiff scaffolds. Control siRNA (si-CTRL) and CAV1 siRNA (si-CAV1) were used. **a** Relative expression levels of adipogenic marker genes and *CAV1*. Data were normalized to that in GM group. *N*= 4. **b**, **c**, **d** Western blot and semi-quantitative analysis of CAV1 and PPAR-γ levels. *N* = 3. **e** Immunofluorescence analysis of CAV1 and C/EBP-α levels. Bar = 100 μm. Oil Red O and BODIPY staining of lipid deposition in cells. Bar = 100 μm. *p<0.05, **p<0.01, ***p<0.001, ****p<0.0001

Similar *CAV1* knockdown studies were conducted in 3D cultures of hBMSCs. Results from qRT-PCR, the western blot and immunofluorescence (Fig. 6a–c, e) showed that CAV1 levels were successfully reduced in 3D culture after siRNA treatment. Reducing CAV1 level resulted in increased expression of adipogenic genes (Fig. 6a), higher protein levels of PPAR-γ (Fig. 6b, d) and C/EBP-α (Fig. 6e), as well as increased oil droplet deposition and BODIPY staining (Fig. 6e) than control counterparts.

### YAP/TAZ pathway is regulated by CAV1

**Fig. 7:**
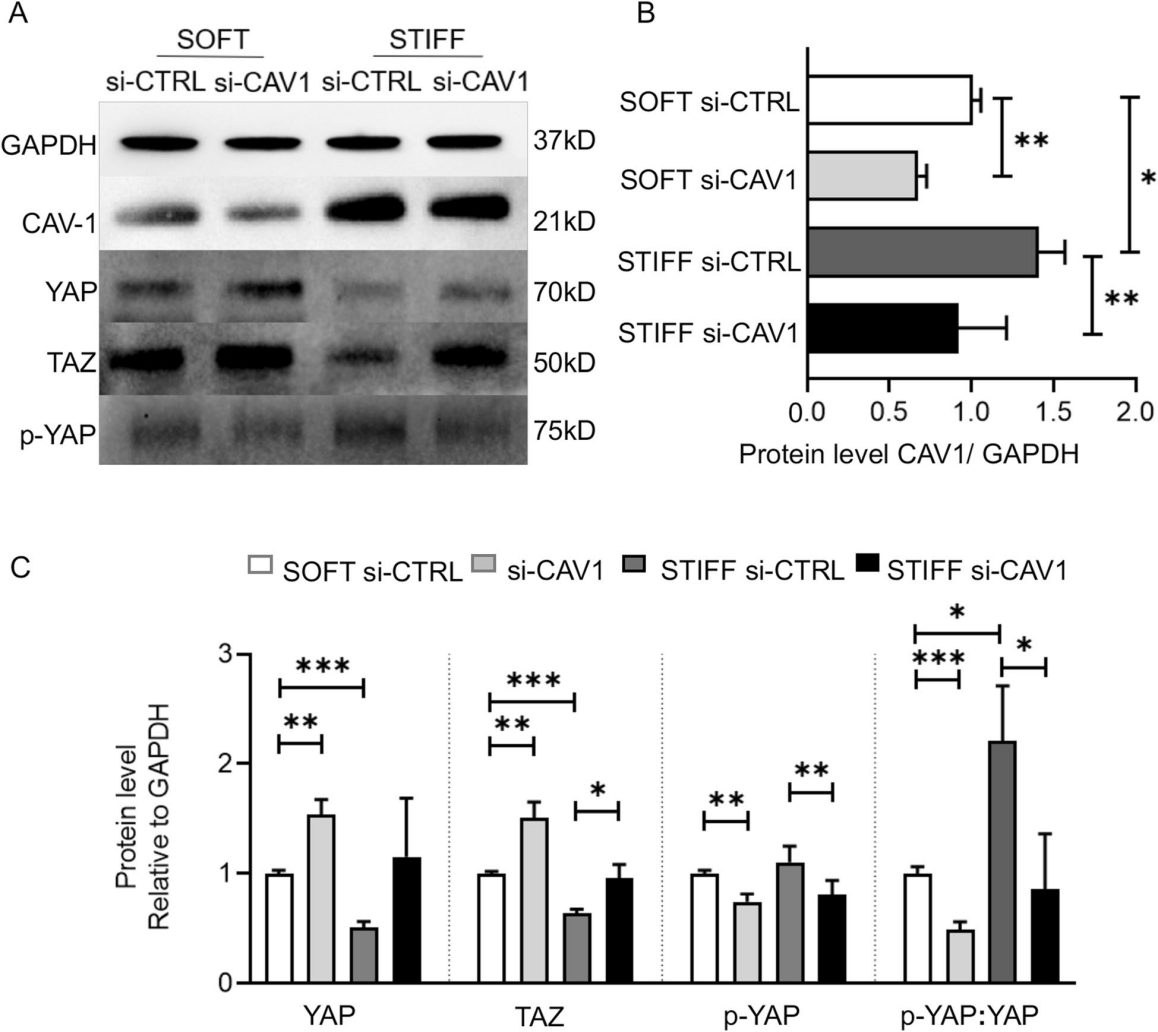
Analysis of CAV1 and YAP/TAZ levels during adipogenesis as a function of scaffold stiffness and treatment with si-CAV1. Cells were treated with si-CTRL or si-CAV1 and then seeded in soft or stiff scaffolds. The samples were collected on day 3 and analyzed by **a** the Western blot to assess protein levels in different groups. Levels of **b** CAV1 and **c** YAP, p-YAP, and TAZ were quantified based on band intensities from Western blots. For each protein analyzed, all values are presented relative to that in soft si-CTRL. *N* = 3. *p<0.05, **p<0.01, ***p<0.001

We then examined the crosstalk between CAV1 and YAP signaling during hBMSC adipogenesis. Western analysis showed that si-CAV1 transfection efficiently reduced CAV1 in cultures maintained in both soft and stiff scaffolds (Fig. 7a, b). Interestingly, in the si-CAV1 group maintained in soft scaffolds (Fig. 7a, c), protein levels of YAP and TAZ rapidly increased and YAP phosphorylation (p-YAP:YAP) decreased, while CAV1 protein levels were reduced (Fig. 7a–c). In contrast, cells within stiff scaffolds displayed higher levels of CAV1, with significantly lower levels of YAP and TAZ, but higher level of YAP phosphorylation (p-YAP:YAP); however, upon si-CAV1 transfection, i.e., with reduction in CAV1 level, YAP and TAZ levels increased, and YAP phosphorylation was reduced (Fig. 7a, c), similar to the response to si-CAV1 treatment seen in the soft scaffold group. These results indicated that YAP/TAZ activation, i.e., unphosphorylated versus phosphorylated YAP, was similarly affected and involved in the modulation of hBMSC adipogenesis as a function of CAV1 level.

## Discussion

Adipose tissue engineering provides a robust tool to regenerate lost or damaged fat. In vitro created adipose tissue has recently been used to model diseases and develop drugs [27, 30–32]. There is thus a demonstrated need for functional fat tissues generated through tissue engineering. In this study, we have induced hBMSCs to undergo adipogenesis within 3D gelatin hydrogels and comparatively analyzed the influence of varying mechanical stiffness of the 3D scaffold in soft supporting adipogenesis. Specifically, we have studied the involvement of CAV1, previously shown to regulate osteogenic differentiation of hBMSCs and the potential role of YAP/TAZ signaling in mediating cell responses to substrate stiffness and adipogenic medium in the context of 3D culture conditions.

**Fig. 8:**
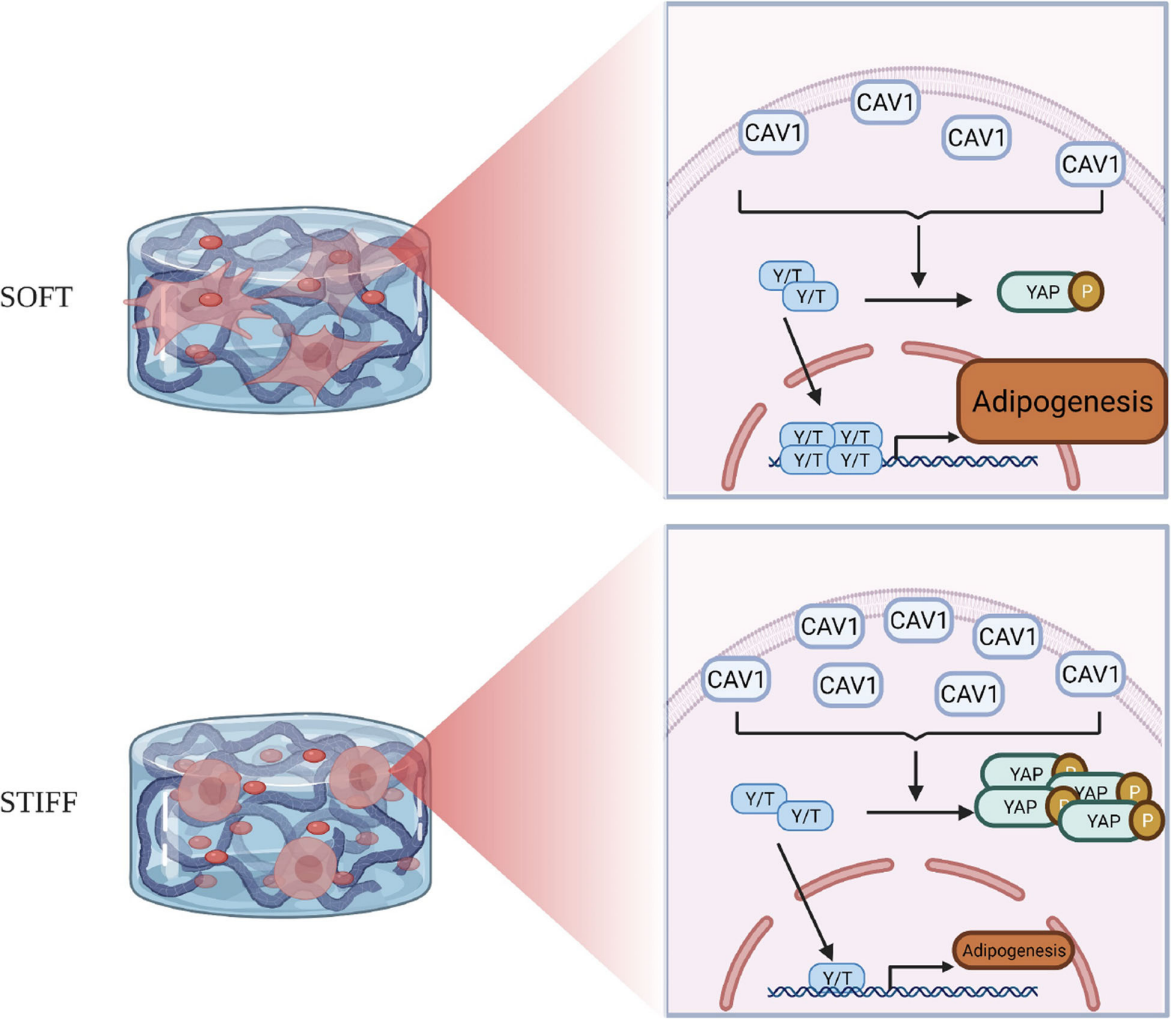
Schematic of coordinated action of CAV1 and YAP/TAZ (Y/T) to regulate hBMSC adipogenesis in soft and stiff scaffolds. Increased stiffness of hydrogel scaffold promotes *CAV1* expression in hBMSCs, thus elevating the phosphorylation of YAP, and reduces the amount as well as translocation of active YAP to nucleus, eventually leading to a lower level of adipogenesis

Based on these observations, we propose how CAV1 and YAP/TAZ act to mediate hBMSC adipogenesis and responses to stiffness of 3D scaffolds (Fig. 8). Specifically, hBMSCs in soft scaffolds display low CAV1 levels, which lead to high expression levels of YAP/TAZ (with corresponding decrease in p-YAP and thus reduced proteasomal degradation of YAP), resulting in subsequent translocation of YAP/TAZ into the nuclei, presumably activating the transcription of adipogenic genes. The si-CAV1-mediated knockdown of *CAV1* further increases the activation of the YAP/TAZ pathway and adipogenesis. Conversely, cells maintained in stiff scaffolds maintain a high level of CAV1, which consequently lead to low activation of YAP/TAZ and higher level of YAP phosphorylation, resulting in reduced adipogenesis.

Given its bioactivities and biocompatibility, gelatin has been widely used in tissue engineering [33]. Its recognized biosafety makes it a convenient biomaterial to generate adipose tissues for different applications. To date, gelatin sponge or gelatin hydrogels have been used to create adipose tissues from human MSCs [32, 34, 35]. In these studies, MSCs displayed reduced adipogenic potential in gelatin cultures when compared to monolayer cultures. Therefore, the optimal conditions that can facilitate MSC adipogenesis within scaffolds, such as those formulated from gelatin, still need to be developed.

It has been well demonstrated that the mechanical properties of substrates, including biomaterial scaffolds, play an important role in mediating MSC differentiation, such as adipogenesis. The first study demonstrating this relationship, in which MSCs were cultured on collagen-coated polyacrylamide gels with different levels of crosslinking and different stiffness characteristics, showed that the softer substrates that mimicked the native stiffness of adipose tissue (2 kPa) resulted in significantly enhanced adipogenesis, compared to harder surface [17]. Similarly, using 3D alginate hydrogel, it was shown that MSCs cultured in a soft scaffold produced more oil droplets and displayed higher expression levels of adipogenic genes than in stiff scaffolds [18]. Currently, it is generally accepted that softer substrates support higher adipogenesis of MSCs regardless of whether they are in 2D or 3D cultures [19]. It should also be noted that the dimensionality of the cultures also affects cell morphology. For example, in 2D cultures, cell spread and extend robustly on a stiff surface or stiff substrate, while in 3D cultures, cells maintain a round shape in stiff scaffolds [21, 36], which was also observed in our study (Fig. 2). These seemingly opposite results raise the question of the importance of cell morphology in guiding MSC differentiation. In a recent review, Zonderland et al. suggested that, on flat 2D substrates, a spread morphology and increased nuclear translocation of YAP are good predictors of MSC lineage commitment such as adipogenic differentiation [37]; however, in 3D culture, these parameters do not have the same predictive power on MSC differentiation. It was further suggested that cell volume might play an important role. For example, large cell volumes benefit adipogenesis. Our findings supported that a spread cell morphology (Fig. 2) along with higher YAP levels (Fig. 4) are good indicators of higher level of adipogenesis in 3D culture as well.

In addition to stiffness, the type and density of ligands present in the extracellular matrix (ECM) also affect the spread of MSCs, a process that requires complicated interactions between receptors on cells and binding ligands in the ECM. For example, the fibronectin tripeptide (RGD) motif is necessary to allow MSCs to spread on a hard surface [38]. However, excessive ECM binding with matrix seems to negatively affect hBMSC adipogenesis on 2D culture. Therefore, to eliminate the influence of different ligand densities, we varied the illumination time for hydrogel photocuring, rather than gelatin concentration, to control the stiffness of photocrosslinkable gelatin [24].

Currently, there is no consensus regarding the functional association and relationship among YAP/TAZ pathway, substrate stiffness, and adipogenesis. Conflicting results have been reported. On 2D culture, increasing YAP activity suppresses adipogenic differentiation [39]. In contrast, inactivation of YAP using small molecule or siRNA promotes adipogenic differentiation [40]. In a study using gelatin scaffolds with stiffness gradients from 8 kPa to 30 kPa, activation of YAP was found in the cells encapsulated in low-stiffness regions [36], similar to our findings here. However, reduced adipogenesis was observed, which is not consistent with our findings (Fig. 3). In another study using polyethylene glycol-based scaffolds, MSCs seeded in soft hydrogels had a larger and more extended cell morphology than those in stiff gels [41]. However, it was found that YAP nuclear function impaired adipogenesis. Moreover, cells exposed to adipogenic stimulation within stiff matrices were shown to displayed higher adipogenesis as compared to those in soft hydrogels. To address this inconsistency, Caliari et al. used the same biomaterials and compared MSC behavior on or within the scaffolds, and showed that increased stiffness promoted cell spreading and YAP/TAZ nuclear localization on 2D culture. However, the opposite trend was observed in cells in 3D culture [21]. Taken together, it is clear that YAP/TAZ signaling is responsible for cell response to stiffness, but the outcome in terms of adipogenesis depends highly on the dimensionality of the culture system.

Interestingly, Khetan et al. demonstrated that the differentiation of MSCs is dictated by the generation of degradation-mediated cellular traction, which was not directly related to cell morphology or matrix mechanics [42]. Specifically, hBMSCs encapsulated within hyaluronic acid hydrogels that permitted cell-mediated degradation exhibited high degrees of adipogenesis. In our study, we have shown that soft hydrogel is more easily degraded by collagenase than its stiff counterpart (Fig. 1b). Of note, a degradable environment is reported to enhance the activity of YAP/TAZ [21], strongly suggesting the importance of matrix degradation in mediating cell behavior in hydrogels.

Although the central role of YAP/TAZ is to dictate MSC response to stiffness, it is not clear how mechanical cues are converted into the biochemical signals that affect this pathway. Previously, we had found that CAV1, a scaffolding protein of cholesterol-rich caveolae lipid rafts in the plasma membrane involved in membrane receptor traffic [43], regulates proliferation and differentiation of hBMSCs [44–46]. We have hypothesized that CAV1 expression may stabilize the differentiated and undifferentiated stem cell phenotype and that changes in CAV1 expression may be required for transition between the two [45]. We thus investigated in this study whether CAV1 also regulates the response of hBMSCs to stiffness. In a recent study investigating fibroblast behavior on soft and hard substrates (2D culture), the activation of YAP in cells cultured on soft substrates was completely abolished when CAV1 was knocked out [23], indicating that CAV1 positively regulates YAP activity. We have obtained the opposite observation here, namely that the suppression of CAV1 results in higher level of YAP (Fig. 7). Our observation is in fact supported by other studies: for example, in the process of mesothelial-to-mesenchymal transition (MMT), the mechanical stretch-activated YAP/TAZ pathway first activates CAV1 expression, then CAV1 conversely suppresses YAP/TAZ [47]. We speculate that the different observations may have come from the different culture conditions (2D versus 3D) used and that CAV1 is unlikely to be the only upstream factor regulating YAP activity. In another study, where CAV1 was overexpressed in hBMSCs, adipogenic differentiation was significantly suppressed [48], consistent with our findings (Fig. 6).

There are some limitations in our study. First, similar to other studies investigating cellular responses to stiffness, this study did not fully eliminate the influence of differing nutrient/waste diffusion rates experienced by the soft and hard scaffolds. However, given the relatively small geometric dimensions of the 3D constructs (2-mm height and 5-mm diameter) and consequently likely limited influence on diffusion related to pore size differences between the soft and stiff scaffolds, this is unlikely to be a major contributing factor. Secondly, whole-cell CAV1 analysis was carried out, and subcellular CAV1 distribution was not done. Namely, we have not analyzed the membrane domain distribution of CAV1, such as association with cholesterol-rich membrane rafts [42, 44], which will be investigated in future studies. Thirdly, our mechanistic study has not included signaling pathways other than Hippo, i.e., YAP/TAZ, that are also known to be related to the cellular response to variations in substrate stiffness, including β-catenin and Rock/Rho pathways [49–51]. Our future mechanistic investigations will include a more in-depth direct comparison of hBMSC behaviors and phenotypes under 2D and 3D cultures, as well as the use of hydrogels that have photo-switchable stiffness to examine the “permanence” of substrate stiffness-mediated cellular signaling.

## Conclusions

Our study demonstrated that softer photocrosslinked 3D gelatin scaffolds promoted more cell spreading and enhanced adipogenesis of encapsulated hBMSCs when compared to more stiff hydrogels. However, the pro-adipogenic effect of the softer scaffold alone was less efficient than that of the adipogenic supplements found in widely used adipogenic culture medium, indicating the importance of biochemical cues. Interestingly, hBMSCs cultured in soft gels displayed reduced levels of CAV1 and increased YAP activity, suggesting CAV1 action upstream of YAP. Given the known involvement of the YAP pathway in mechanosensing of substrate stiffness and its complex downstream signaling pathway, which is also modulated as a function of the dimensionality of the culture system, our findings reported here suggest that suppressing CAV1 expression is likely an efficient way to enhance adipogenesis. The 3D culture models developed here also provide a robust tool to study other behaviors of MSCs in response to stiffness, such as osteogenesis, neurogenesis, and chondrogenesis.

## Supplementary Information

**Additional file 1: Figure S1**. Relative expression levels of adipogenic marker genes in hBMSCs cultured under adipogenic conditions (AM) in soft or stiff scaffolds. Data are normalized to that from Soft-GM group (cultured under control growth conditions). N = 4. *, p<0.05; **, p<0.01; ****, p<0.0001. GM = growth medium; AM = adipogenic medium. **Figure S2**. Relative expression level of CAV1 at 48 h after transfection. Data are normalized to that in CTRL group, which was not treated with siRNA or transfection agents. N = 4. ***, p<0.001; ****, p<0.0001. **Table S1**. Primer sequences for qRT-qPCR. **Table S2**. Information of antibodies used in this study.

## Data Availability

The data sets supporting the conclusions of this article are included within the article and its supplementary files.

## Abbreviations

hBMSCs: Human bone marrow-derived mesenchymal stem cells
CAV1: Caveolin-1
YAP/TAZ: Yes-associated protein/Tafazzin
siRNA: Small interfering RNA
qRT-PCR: Quantitative real-time polymerase chain reaction
2D: Two-dimensional
3D: Three-dimensional
GelMA: Methacrylated gelatin
LAP: Lithium phenyl-2,4,6-trimethyl-benzoyl phosphinate
PBS: Phosphate-buffered saline
PBS-T: Phosphate-buffered saline-Tween
PFA: Paraformaldehyde
GM: Growth medium
AM: Adipogenic medium
RIPA: Radioimmunoprecipitation assay
PVDF: Polyvinylidene fluoride
HRP: Horseradish peroxidase
