# Supplementary material for: Caveolin-1 mediates soft scaffold-enhanced adipogenesis of human mesenchymal stem cells
Source: Stem Cell Res Ther. 2021 Jun 14;12:347. doi: 10.1186/s13287-021-02356-z (PMC8201886; doi:10.1186/s13287-021-02356-z)
Supplement: Supplementary file 3 — Additional file 1: Figure S1. Relative expression levels of adipogenic marker genes in hBMSCs cultured under adipogenic conditions (AM) in soft or stiff scaffolds. Data are normalized to that from Soft-GM group (cultured under control growth conditions). N = 4. *, p<0.05; **, p<0.01; ****, p<0.0001. GM = growth medium; AM = adipogenic medium. Figure S2. Relative expression level of CAV1 at 48 h after transfection. Data are normalized to that in CTRL group, which was not treated with siRNA or transfection agents. N = 4. ***, p<0.001; ****, p<0.0001. Table S1. Primer sequences for qRT-qPCR. Table S2. Information of antibodies used in this study. [file 13287_2021_2356_MOESM1_ESM.docx]

**Supplementary Material**

**Caveolin-1 Mediates Soft Scaffold-enhanced Adipogenesis of**

**Human Mesenchymal Stem Cells**

Shiqi Xiang^1,2^, Zhong Li^1^, Madalyn R. Fritch^1^, La Li^1^, Sachin Velankar^3^, Yuwei Liu^1^,

Jihee Sohn^1,#^, Natasha Baker^1,†^, Hang Lin^1,4,5,*^, Rocky S. Tuan^1,4,5,‡,*^

^1^Center for Cellular and Molecular Engineering, Department of Orthopaedic Surgery,

University of Pittsburgh School of Medicine, Pittsburgh, Pennsylvania, USA

^2^Department of Orthopedics, The Second Xiangya Hospital,

Central South University, Hunan, PR China

^3^Department of Chem/Petroleum Engineering and Mechanical Engineering & Materials Science, University of Pittsburgh Swanson School of Engineering, Pittsburgh, Pennsylvania, USA.

^4^McGowan Institute for Regenerative Medicine, University of Pittsburgh, Pittsburgh, Pennsylvania, USA

^5^Department of Bioengineering, University of Pittsburgh Swanson School of Engineering, Pittsburgh, Pennsylvania, USA.

***Correspondence**:

Hang Lin, email: hal46@pitt.edu

Rocky S. Tuan, email: tuanr@cuhk.edu.hk

**Current addresses**:

^#^Biogen, Boston, Massachusetts, USA

^†^Department of Oral Biology, University of Pittsburgh School of Dental Medicine, Pittsburgh, Pennsylvania, USA

^‡^The Chinese University of Hong Kong, Institute for Tissue Engineering and Regenerative Medicine, Shatin, Hong Kong SAR, China

**Figure S1**


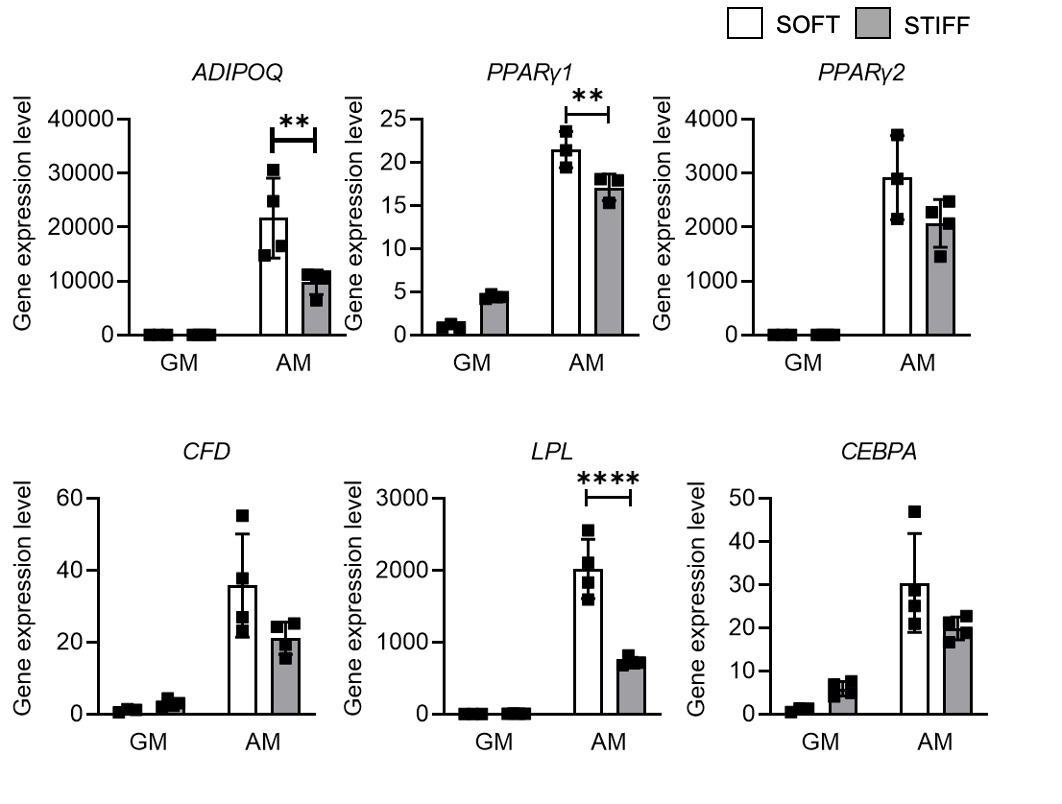


**Figure S1.** Relative expression levels of adipogenic marker genes in hBMSCs cultured under adipogenic conditions (AM) in soft or stiff scaffolds. Data are normalized to that from Soft-GM group (cultured under control growth conditions). N = 4. *, p<0.05; **, p<0.01; ****, p<0.0001. GM = growth medium; AM = adipogenic medium.

**Figure S2**


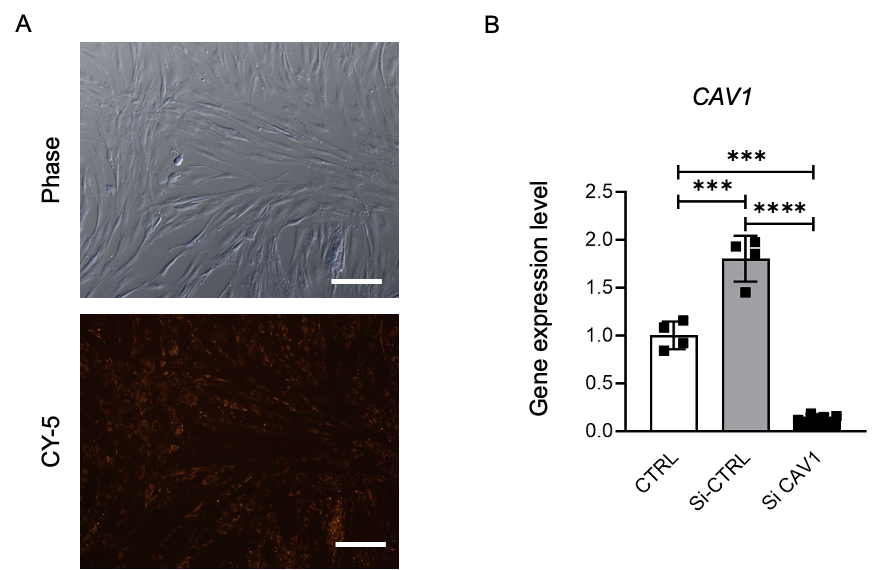


**Figure S2.** Relative expression level of *CAV1* at 48 h after transfection. Data are normalized to that in CTRL group, which was not treated with siRNA or transfection agents. N = 4. ***, p<0.001; ****, p<0.0001.

**Table S1**

**Primer sequences for qRT-qPCR**

| Gene | Forward primer (5’→3’) | Reverse primer (5’→3’) |
| --- | --- | --- |
| *GAPDH* | CAAGGCTGAGAACGGGAAGC | AGGGGGCAGAGATGATGACC |
| *CFD* | AACATGCCCATTCGCTTTAC | AGAGGCTGACCTTCACATCC |
| *LPL* | GAGATTTCTCTGTATGGCACTG | CTGCAAATGAGACACTTTCTC |
| *PPARγ1* | GACAGGAAAGACAACAGACAAAT | GGGGTGATGTGTTTGAACTTG |
| *PPARγ2* | TCCATGCTGTTATGGGTGAA | TGTGTCAACCATGGTCATTTC |
| *CEBPA* | GACTTCTACGAGGCGGAGC | AGGTGGGGCGGGAGG |
| *CAV1* | GGGCAACATCTACAAGCCCAACAA | CTGATGCACTGAATCTCAATCAGGAA |
| *CFD* | GACACCATCGACCACGACC | GCCACGTCGCAGAGAGTTC |

**Table S2**

**Information of antibodies used in this study**

| **Method** | **Antibodies** | **Company** | **Catalog No.** | **Dilution** |
| --- | --- | --- | --- | --- |
| Immunostaining | CAV1 | Cell Signaling Technology | 3267s | 1:400 |
|  | C/EBP-α | Cell Signaling Technology | 8178s | 1:100 |
|  | Goat Anti-Rabbit IgG H&L (Alexa Fluor 488) | Abcam | ab150077 | 1:500 |
| Western blot | GAPDH | Cell Signaling Technology | 5174s | 1:2000 |
|  | Rabbit IgG HRP Linked Whole Ab | Sigma-Aldrich | GENA934-1ML | 1:2000 |
|  | CAV1 | Cell Signaling Technology | 3267s | 1:1000 |
|  | C/EBP-α | Cell Signaling Technology | 8178s | 1:1000 |
|  | PPARγ | Cell Signaling Technology | 2430s | 1:1000 |
|  | Phospho-YAP (Ser127) | Cell Signaling Technology | 13008s | 1:1000 |
|  | YAP/TAZ | Cell Signaling Technology | 8418s | 1:1000 |
|  | YAP | Cell Signaling Technology | 14074s | 1:1000 |
|  | Phospho-YAP (Ser397) | Cell Signaling Technology | 13619s | 1:1000 |
